# Four-corner fusion method using a bioabsorbable plate for scapholunate advanced collapse and scaphoid nonunion advanced collapse wrists: a case series study

**DOI:** 10.1186/s12891-020-03709-0

**Published:** 2020-10-15

**Authors:** Yukichi Zenke, Toshihisa Oshige, Kunitaka Menuki, Hideyuki Hirasawa, Yoshiaki Yamanaka, Takafumi Tajima, Kenji Kosugi, Akinori Sakai

**Affiliations:** 1 Department of Orthopaedic Surgery, University of Occupational and Environmental Health, 807-8555 Iseigaoka Yahatanisiku, Kitakyusyu City, Fukuoka Japan; 2 Department of Orthopaedic Surgery, Tobata Kyoritsu Hospital, Kitakyushu, Japan; 3 Department of Orthopaedic Surgery, Tokyo Rosai Hospital, Tokyo, Japan

**Keywords:** Bioabsorbable plate, Four-corner fusion, Scapholunate advanced collapse, Scaphoid nonunion advanced collapse

## Abstract

**Background:**

Scaphoid excision and four-corner arthrodesis is an acceptable salvage procedure for the treatment of scapholunate advanced collapse (SLAC) and scaphoid nonunion advanced collapse (SNAC) wrists, since first popularized in the 1980s. We investigated the potential application of novel bioabsorbable plates and screws made of un-sintered hydroxyapatite/poly-L-lactide composite for the treatment of metacarpal fractures. We used this material for the fixation of four-corner fusions for SLAC or SNAC wrists commencing from April 2009. The purpose of this study was to clarify the controversy in the literature regarding the use of these plates.

**Methods:**

The surgical procedures and clinical outcomes of four-corner fusions using a bioabsorbable (poly-L-lactic acid and hydroxyapatite) plate were reported. Ten patients (mean age, 59.2 years) with SLAC or SNAC wrists underwent fusions between April 2009 and June 2016. The primary diseases were scapholunate ligament injury, Preiser disease, and scaphoid pseudarthrosis. The mean postoperative follow-up period was 45.9 months (range, 12–86).

**Results:**

In all patients, bone union was achieved without dislocation or pain. The mean wrist flexion and extension arc improved from 78.5 degrees before surgery to 90.5 degrees after surgery. Mean grip strength improved from 51 to 69% after surgery, and the Quick Disabilities of the Arm, Shoulder, and Hand (*Quick*DASH) score improved from 53.5 to 14.3. No complications such as infection, avascular swelling, or tendon adhesion were observed. This implant requires no removal of internal fixation devices, produces stable outcomes, and is an effective fusion technique.

**Conclusions:**

We summarized the outcomes of four-corner arthrodesis using bioabsorbable plates. Satisfactory clinical results were shown, with no obvious complications. This novel plate also serves as a good alternative for patients who are allergic to metals. Furthermore, bioabsorbable plates are advantageous as they do not require removal.

## Background

Scaphoid excision and four-corner arthrodesis (FCA) is an acceptable salvage procedure for the treatment of scapholunate advanced collapse (SLAC) and scaphoid nonunion advanced collapse (SNAC) since it was first popularized by Watson and Ballet in 1984 [1] and Vender and Watson in 1987 [2]. This arthrodesis traditionally involves the use of Kirschner wires [1, 2], staples [3, 4], or compression screws [5] to achieve fixation of the lunate, capitate, hamate, and triquetrum. In 1999, dorsal circular plates (DCPs) were developed to allow for earlier range of motion, while limiting postoperative stiffness and nonunion; this was to address nonunion rates as high as 63% [6]. The first DCP designed for FCA (Spider TM Limited Wrist Fusion Plate [Kinetikos Medical Inc., San Diego, CA, USA]), resulted in higher rates of fusion and better functional outcomes than anticipated [7]. More recently, locking DCPs have been introduced [8]. The Xpode® Cup (TriMed Inc., Santa Clarita, CA) was the first in this class to be composed of a radiolucent polyether-ether-ketone (PEEK-Optima) [9].

In the current study, we investigated the potential application of novel bioabsorbable plates (BAPs) and screws made of un-sintered hydroxyapatite/poly-L-lactide (u-HA/PLLA) composite for the treatment of metacarpal fractures [10]. We used this material for the fixation of four-corner fusions for SLAC or SNAC wrists commencing from April 2009. The purpose of this study was to clarify the controversy in the literature regarding use of these plates. We therefore reviewed our experience of scaphoid excision four-corner fusions using a BAP to 1) determine the union rate of the four bones, 2) identify the surgical complications, and 3) determine the functional outcomes. The functional outcomes included active range of motion (ROM), grip strength (GS), *Quick*DASH score [11], and patient evaluated pain grade.

## Methods

We retrospectively reviewed the medical records and radiographs of ten patients who were surgically treated for radioscaphoid arthritis with FCA using a BAP (Super Fixorb MX40; Johnson & Johnson and Takiron, Tokyo) between 2009 and 2016. The BAP is comprised of u-HA (40% w/w) and PLLA (60% w/w) composite. The surgical indications were SLAC wrist in three patients and SNAC wrist in seven. There were five female and five male patients, with a mean age of 59.2 years (range, 33–71). In terms of comorbidities, one patient had noninsulin-dependent diabetes, two patients had hypertension, one had iron-deficiency anemia, and two had gastroesophageal reflux disease. The mean period from onset of pain to surgery was 46.3 months (range, 2–240 months), and the minimum follow up was 12 months (mean, 45.9 months; range, 12–86) (Table 1). No patients were recalled specifically for this study. There were no contraindications of this method for four-corner fusion. Institutional Review Board approval was obtained for this study. In addition, the consent for this study was obtained for each patient.

**Table 1:** Summary of patients surgically treated for SLAC & SNAC wrists

Case	Sex	Age, years	Side	Occupation	From onset, months	Stage	Final follow up	
**1**	**F**	**65**	**R**	**House wife**	**240**	**2**	**86**	**SNAC**
**2**	**F**	**69**	**L**	**House wife**	**5**	**2**	**67**	**SNAC**
**3**	**M**	**48**	**L**	**Manufacturer**	**72**	**3**	**12**	**SNAC**
**4**	**M**	**54**	**L**	**Manufacturer**	**7**	**2**	**86**	**SNAC**
**5**	**M**	**56**	**R**	**Farmer**	**60**	**3**	**60**	**SNAC**
**6**	**F**	**66**	**L**	**Farmer**	**6**	**2**	**58**	**SLAC**
**7**	**M**	**71**	**R**	**Potter**	**2**	**3**	**19**	**SLAC**
**8**	**M**	**69**	**R**	**Driver**	**12**	**3**	**18**	**SNAC**
**9**	**F**	**33**	**L**	**None**	**36**	**3**	**36**	**SLAC**
**10**	**F**	**61**	**R**	**None**	**16**	**3**	**17**	**SNAC**
**Mean**		**59.2**			**46.3**		**45.9**	

*F* Female; *L* Left; *M* Male; *R* Right; *SLAC* scapholunate advanced collapse; *SNAC* scaphoid nonunion advanced collapse

### Technique

**Fig. 1:**
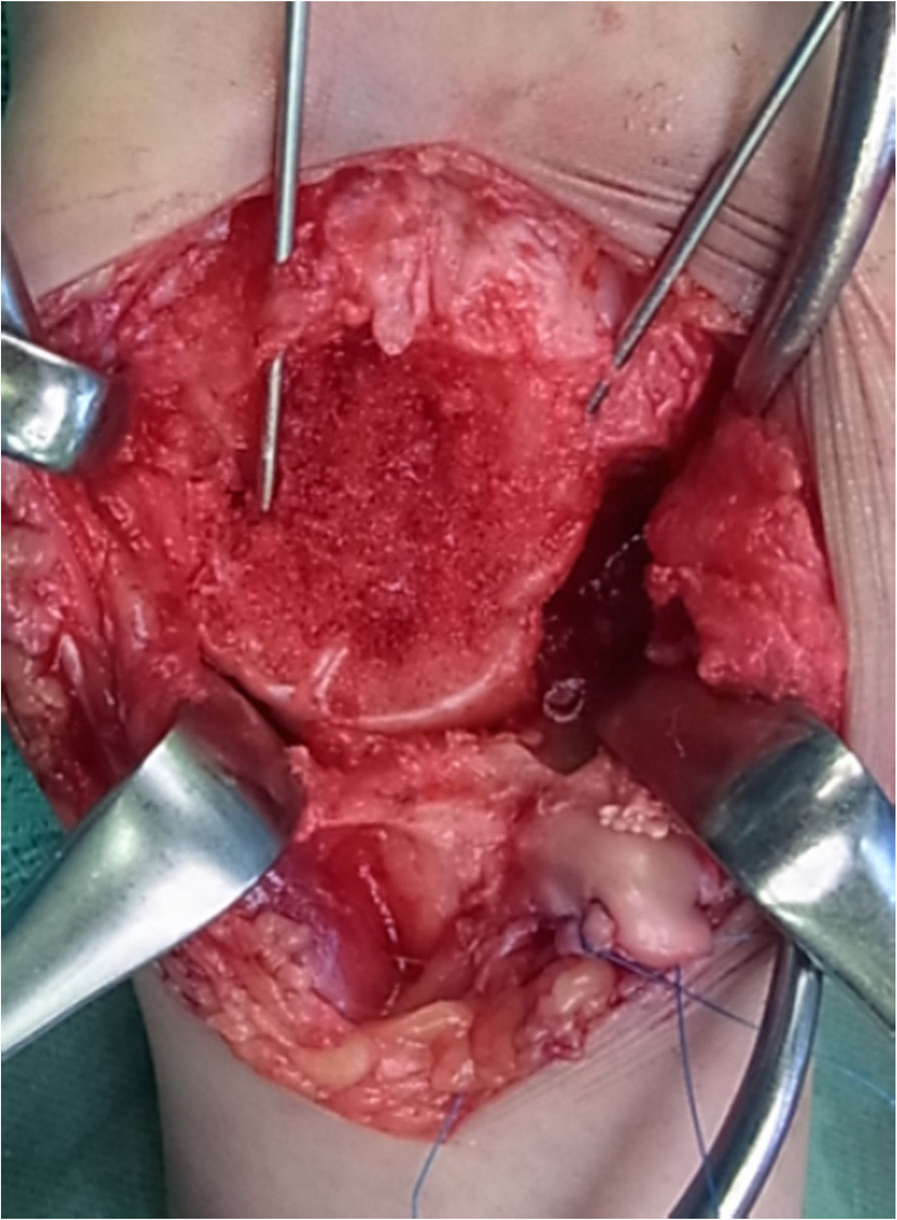
Two additional Kirschner wires (from the dorsal side) maintain the reduced position

The surgical technique is described in Additional File 1. We performed each surgery through a dorsal midline incision. The extensor pollicis longus tendon was released and transposed radially. The dorsal capsule was incised longitudinally and partially elevated from the dorsal cortex of the distal radius. We segmentally excised the posterior interosseous nerve. The scaphoid was excised in a piecemeal fashion using an osteotome and bone rongeur. We used a small curette to remove all remaining articular cartilage and eburnated subchondral bone along the fusion surfaces. The dorsi-flexed lunate was realigned by hyper flexing the wrist. A 1.5-mm smooth Kirschner wire was driven along the dorsal edge of the distal radial articular surface into the lunate to maintain the position. We placed at least two additional Kirschner wires (from the dorsal side) to maintain the reduced position of the four-corner carpal bones (Fig. 1). Fluoroscopy was utilized to confirm the position of the bones.

**Fig. 2:**
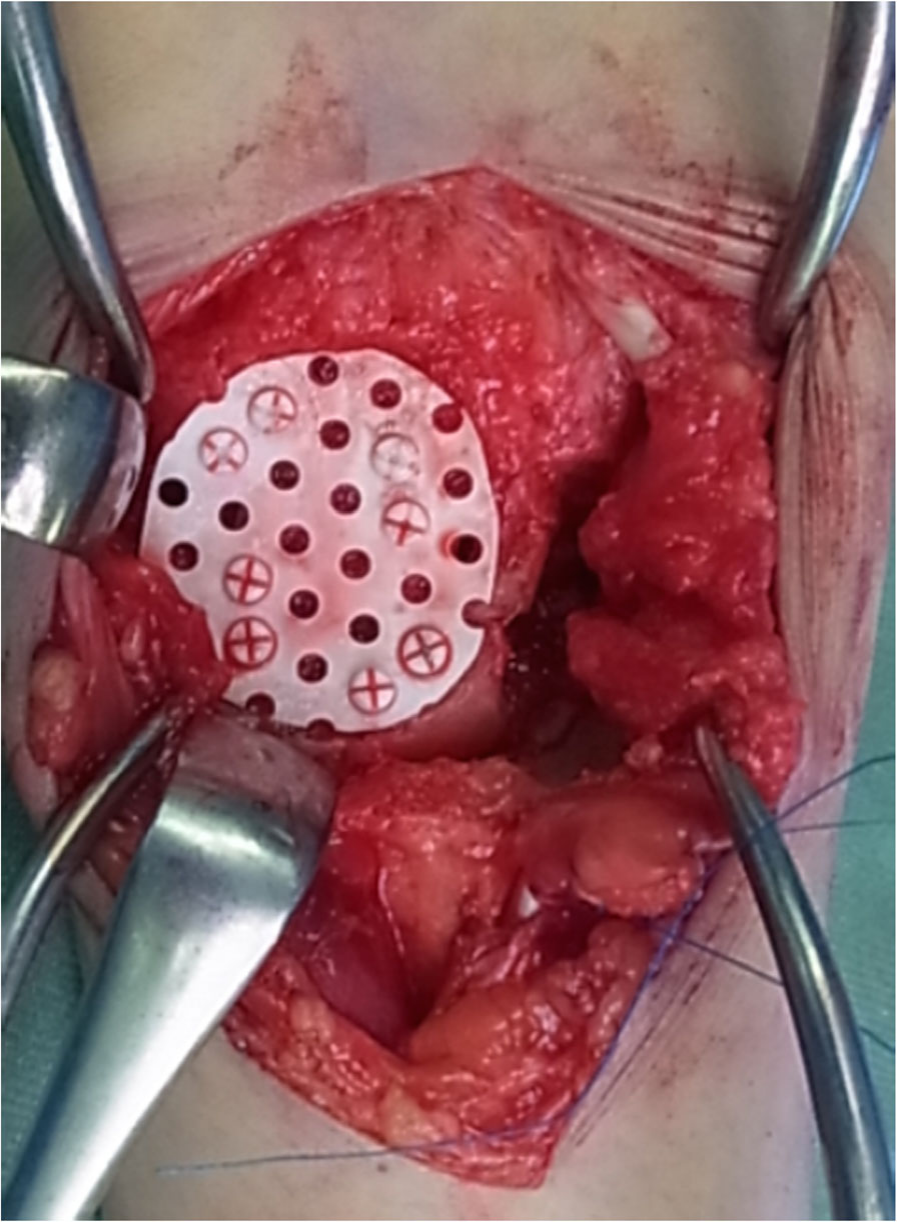
A circle-shaped, self-crafted BAP was used, and we inserted 2.0-mm bioabsorbable screws for fixation. BAP, bioabsorbable plate

**Fig. 3:**
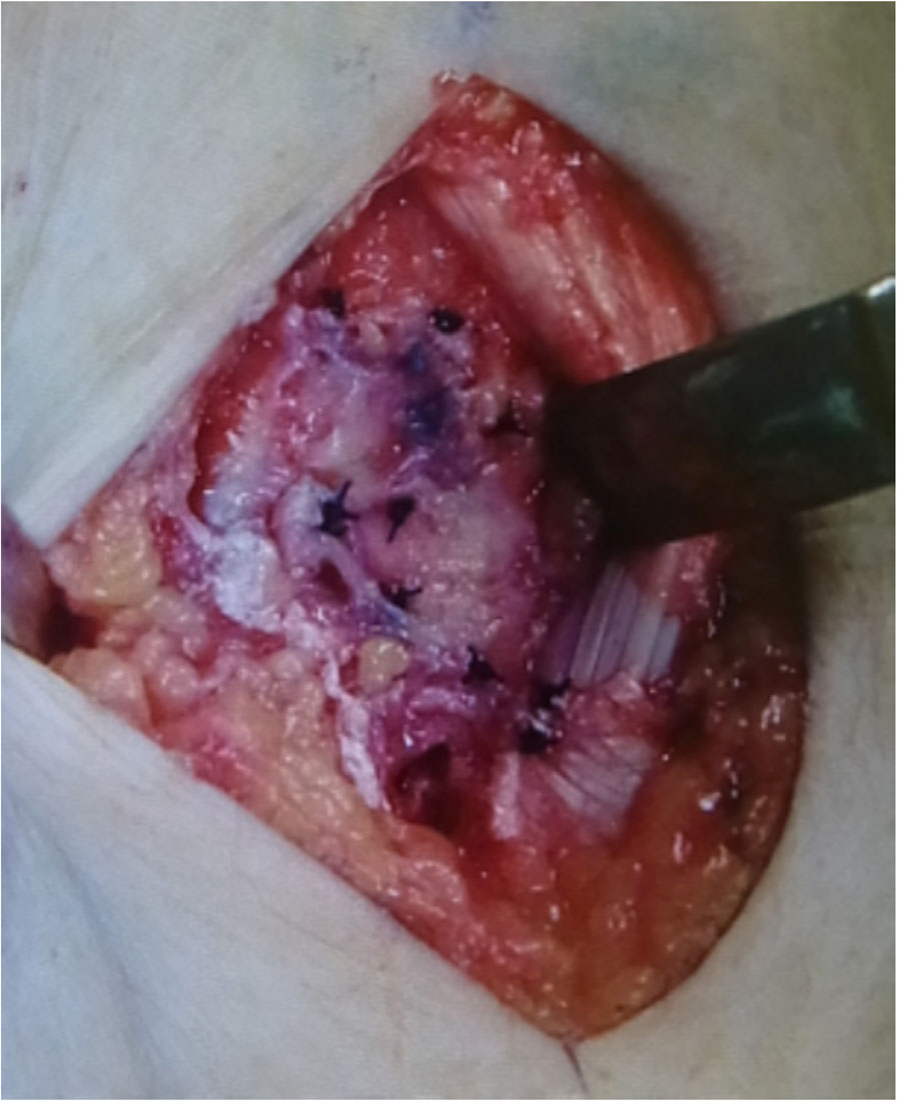
After removal of the Kirschner wires, we repaired the capsule and skin closure was completed

In all cases, we harvested a cancellous bone graft from the ipsilateral iliac bone through a small skin incision using a curved curette. No radial styloidectomies were performed. A Surgairtome was then used to prepare the BAP fixation surface. We reamed until the reamer edge was seated below the dorsal cortex of the lunate. Cancellous bone graft was tightly packed between the fusion surfaces and within the intercarpal bone area (Fig. 1). In all cases, a circle-shaped, self-crafted BAP was used, and we inserted 2.0-mm bioabsorbable screws for fixation (Fig. 2). The center of the plate was packed with additional bone graft. Fluoroscopy was again used to confirm appropriate alignment of the bones and the length of the screws. Passive extension of the wrist was performed to assess dorsal hardware impingement clinically and radiographically. After removal of the Kirschner wires, we repaired the capsule, and skin closure was completed (Fig. 3).

### Postoperative treatment

A plaster forearm-based thumb Spica splint was applied to decrease swelling and was maintained for 1–2 weeks. This was followed by a fiberglass wrist splint for an additional 2 weeks. All patients received a minimum of 8 weeks of occupational therapy afterwards. Supervised sessions with an occupational therapist took place twice a week, and an at-home therapy program was performed by the patient every day. Patients were seen monthly until they were discharged.

### Evaluations

We recorded objective measurements such as the GS (Jamar dynamometer in the second handle position), wrist active ROM, dorsi-flexion (DF), and volar-flexion (VF) (using a goniometer). These measurements were compared with those of the opposite unaffected wrist. All patients were asymptomatic in their contralateral wrists and hands. We asked patients to subjectively grade their level of pain as 1 = none; 2 = slight (minimal with activity, none at rest); 3 = moderate (moderate with activity, occasional at rest); or 4 = severe (constant and significant). For a comparison with the literature, we used the *Quick*DASH score to assess fusion, pain, ROM, and GS.

Radiographs were taken immediately postoperatively and then at 2 weeks, 4 weeks, 8 weeks, 12 weeks, 6 months, and at the final postoperative follow up. The treating surgeons used radiographs to confirm successful fusion, to rule out hardware complications, and to assess signs of nonunion. Successful arthrodesis was determined by solid trabeculation across the intercarpal articulations and no persistent joint lines visible on the radiographs. The radiograph was inspected for any lucency around the implant, implant failure or screw backout, joint-line narrowing, marginal osteophytes, subchondral cystic changes, and carpal malalignment.

## Results

**Fig. 4:**
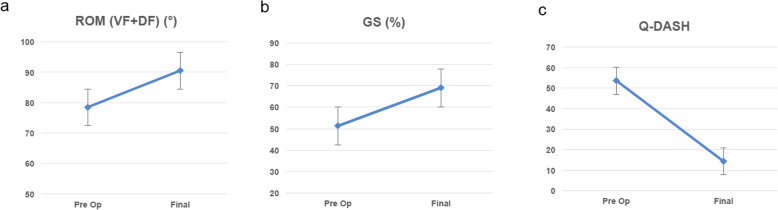
Final range of motion (**a**), GS (**b**), and *Quick*DASH (**c**) at final follow up. GS, grip strength; *Quick*DASH, Quick Disabilities of the Arm, Shoulder, and Hand

The clinical outcomes are shown in Table 2. The preoperative mean ROM (DF + VF) was 78.5^°^ (standard deviation [SD], 21.7; range, 40–120). The final ROM was 90.5^°^ (SD, 21.1; range, 60–130) (Fig. 4a). Preoperative mean GS was 51% (SD, 22.3; range, 16.7–77.8) of the contralateral value and 69% (SD, 16.5; range, 47.6–104.7) at final follow up (Fig. 4b). The mean *Quick*DASH score was 53.5 (SD, 19.8; range, 29.5–81.8) preoperatively and 15.1 (SD, 10.8; range, 0–38.6) at the final follow up (Fig. 4c). The mean duration off work was 4.5 months (SD, 3.8; range, 3–9) and 90% of the patients were able to return to work. Two patients required job adaptation. Seven patients reported no pain, two patients reported slight pain, and one patient reported moderate pain at the final follow up. No patients reported severe pain and nine patients expressed satisfaction with the operation. Two of the case results are included below as they are of particular interest to the study.

**Table 2:** Clinical outcomes

	Age	Preoperative					Final					Pain Grade
		VF (°)	DF (°)	VF + DF (°)	GS (%)	***Quick***DASH	VF (°)	DF (°)	VF + DF (°)	GS (%)	***Quick***DASH	
**1**	**65**	**60**	**30**	**90**	**72.8**	**31.8**	**60**	**40**	**100**	**70.6**	**9.09**	**None**
**2**	**69**	**40**	**60**	**100**	**68.3**	**34.1**	**60**	**40**	**100**	**47.6**	**9.09**	**None**
**3**	**48**	**50**	**70**	**120**	**74.4**	**29.5**	**50**	**40**	**90**	**67.2**	**0**	**None**
**4**	**54**	**40**	**50**	**90**	**37.3**	**61.4**	**60**	**70**	**130**	**104.7**	**6.82**	**None**
**5**	**56**	**10**	**30**	**40**	**53.3**	**72.7**	**35**	**60**	**95**	**53.4**	**22.7**	**Slight**
**6**	**66**	**30**	**30**	**60**	**49.7**	**40.9**	**40**	**30**	**70**	**61.6**	**15.9**	**None**
**7**	**71**	**40**	**30**	**70**	**16.7**	**81.8**	**40**	**20**	**60**	**72.1**	**18.2**	**None**
**8**	**69**	**30**	**30**	**60**	**19.9**	**59.1**	**30**	**50**	**80**	**67**	**15.9**	**Slight**
**9**	**33**	**30**	**45**	**75**	**42.1**	**70.5**	**20**	**50**	**70**	**59**	**38.6**	**Moderate**
**10**	**61**	**30**	**50**	**80**	**77.8**	**No data**	**40**	**70**	**110**	**86.6**	**6.82**	**None**
**AVG**	**59.2**	**36**	**42.5**	**78.5**	**51.2**	**53.5**	**43.5**	**47**	**90.5**	**69.0**	**15.1**	
**SD**	**11.9**	**13.5**	**14.8**	**21.7**	**22.3**	**19.8**	**13.8**	**16.4**	**21.1**	**16.5**	**10.8**	

*AVE* Average; *SD* Standard Deviation; *VF* volar-flexion; *DF* dorsi-flexion; *GS* grip strength; *QuickDASH* Quick Disabilities of the Arm, Shoulder, and Hand

### Complications

No patients underwent a secondary total fusion operation. Complications included two nonunions or fibrous unions on the radiographs (union rate: 80%). However, neither required revision by bone graft, and no delayed union was observed after 3 months. No cases required revision surgery and all patients became asymptomatic.

In a single case, the patient (a farmer) ceased all severe stresses that he had subjected to his wrist between the 6th and 12th postoperative months; he then became asymptomatic. In all cases, a computed tomography scan was performed to assess bone healing, as the radiographic images were inconclusive. There were no cases of material related symptomatic dorsal impingement or aseptic necrosis. In a single case, slight progressive destruction of the four corners, and loss of carpal height, were observed. The patient remained asymptomatic and no further action has been taken to date.

There were two instances of the implant breaking; however, the patients were asymptomatic and only elicited radiological changes.

### Representative cases

**Fig. 5:**
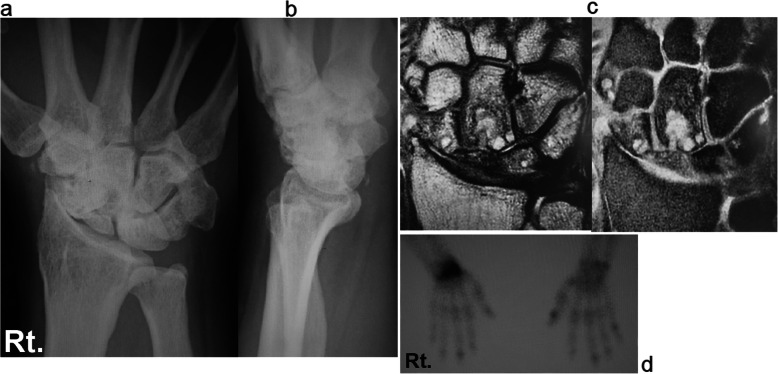
Radiographs (**a**, **b**), computed tomography scan (**c**), and bone scintigram (**d**) of a 56-year-old farmer. The patient suffered from radioscaphoid and capitolunate osteoarthritis of the right wrist caused by SNAC stage III following a fall 5 years ago. SNAC, scaphoid nonunion advanced collapse

**Fig. 6:**
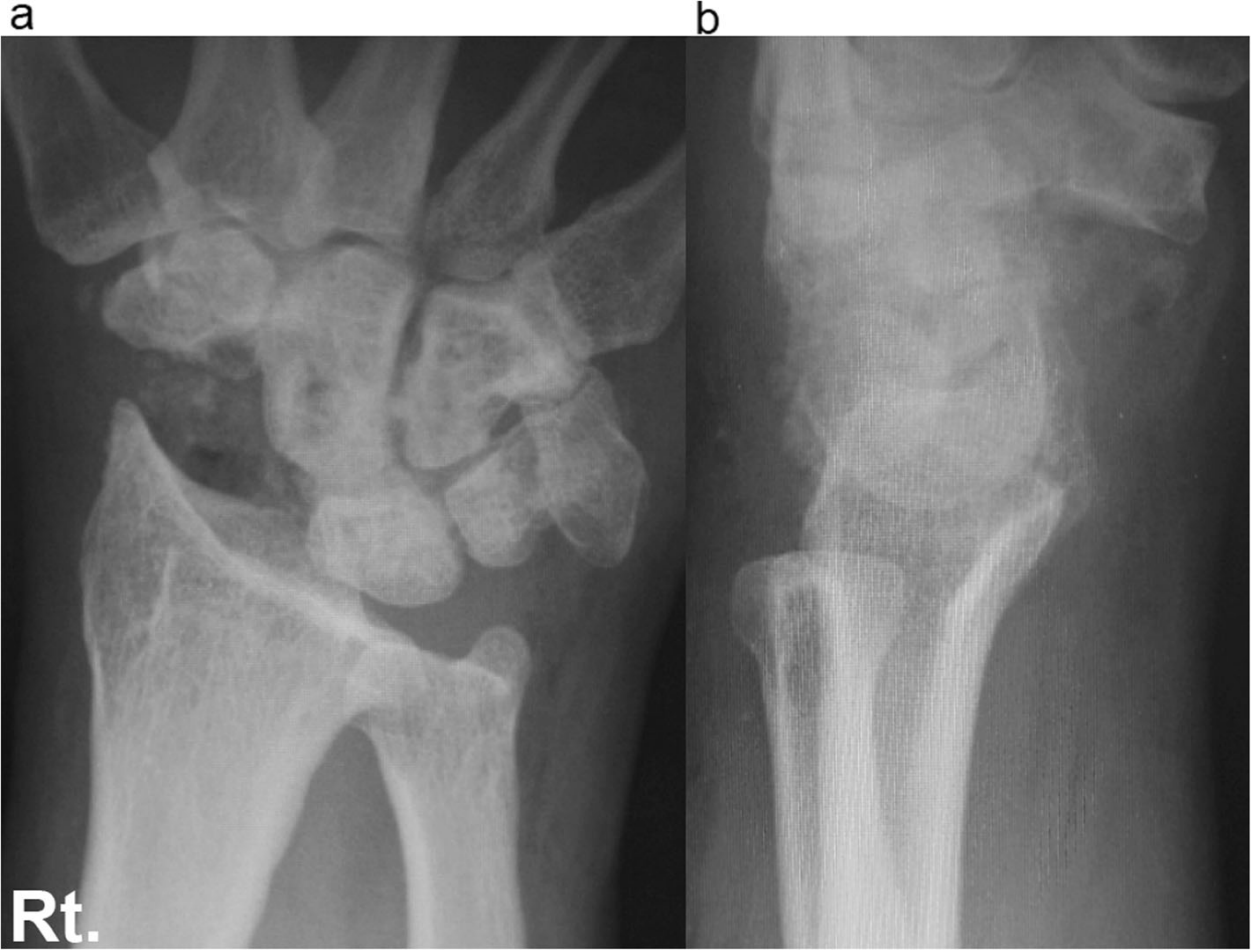
Postoperative X-rays of a 56-year-old male at the one-year follow up

**Fig. 7:**
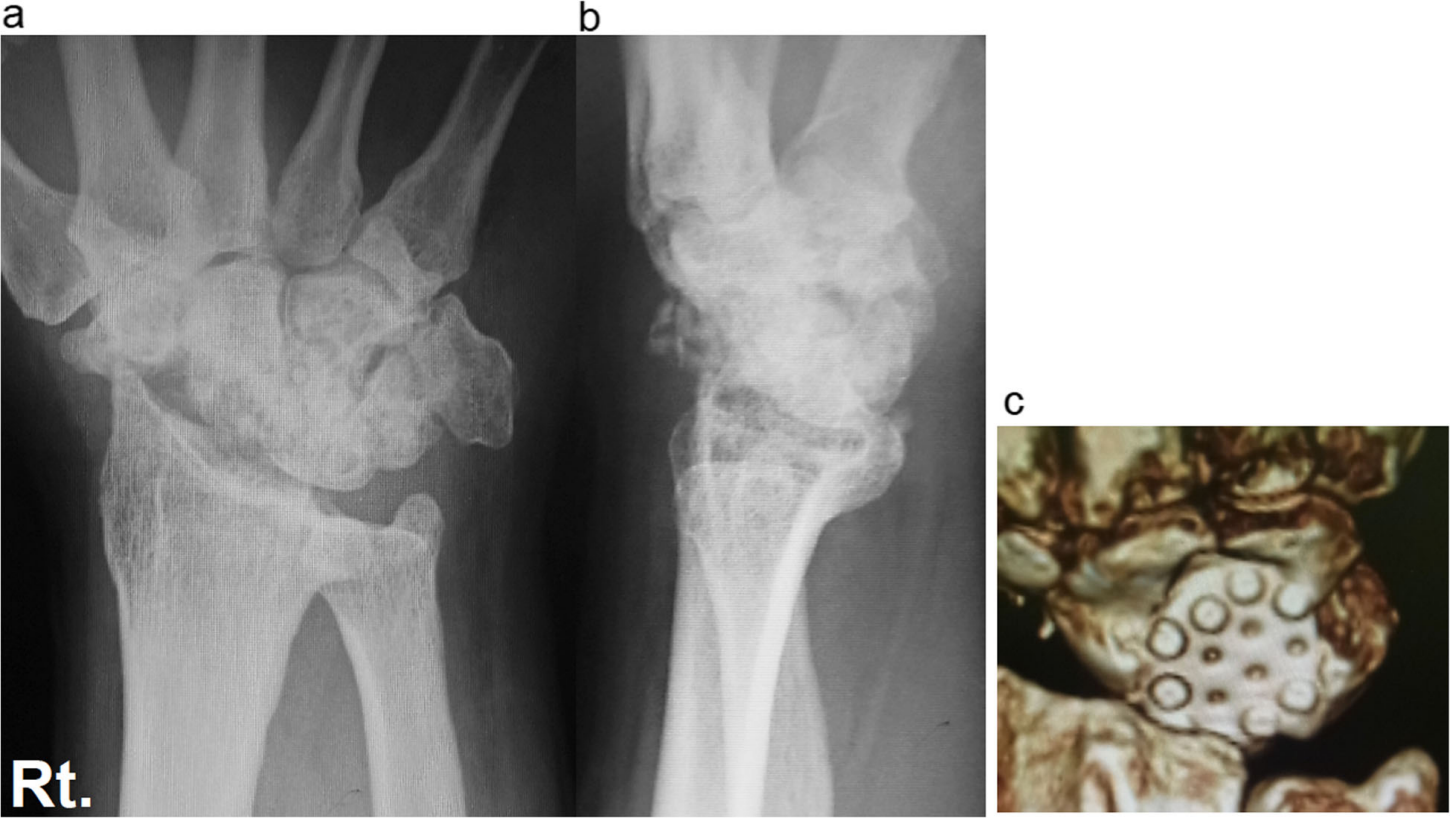
Postoperative X-rays (**a**, **b**) and three-dimensional computed tomography scan (**c**). Fifty-six-year-old male patient at the one-year follow up

**Fig. 8:**
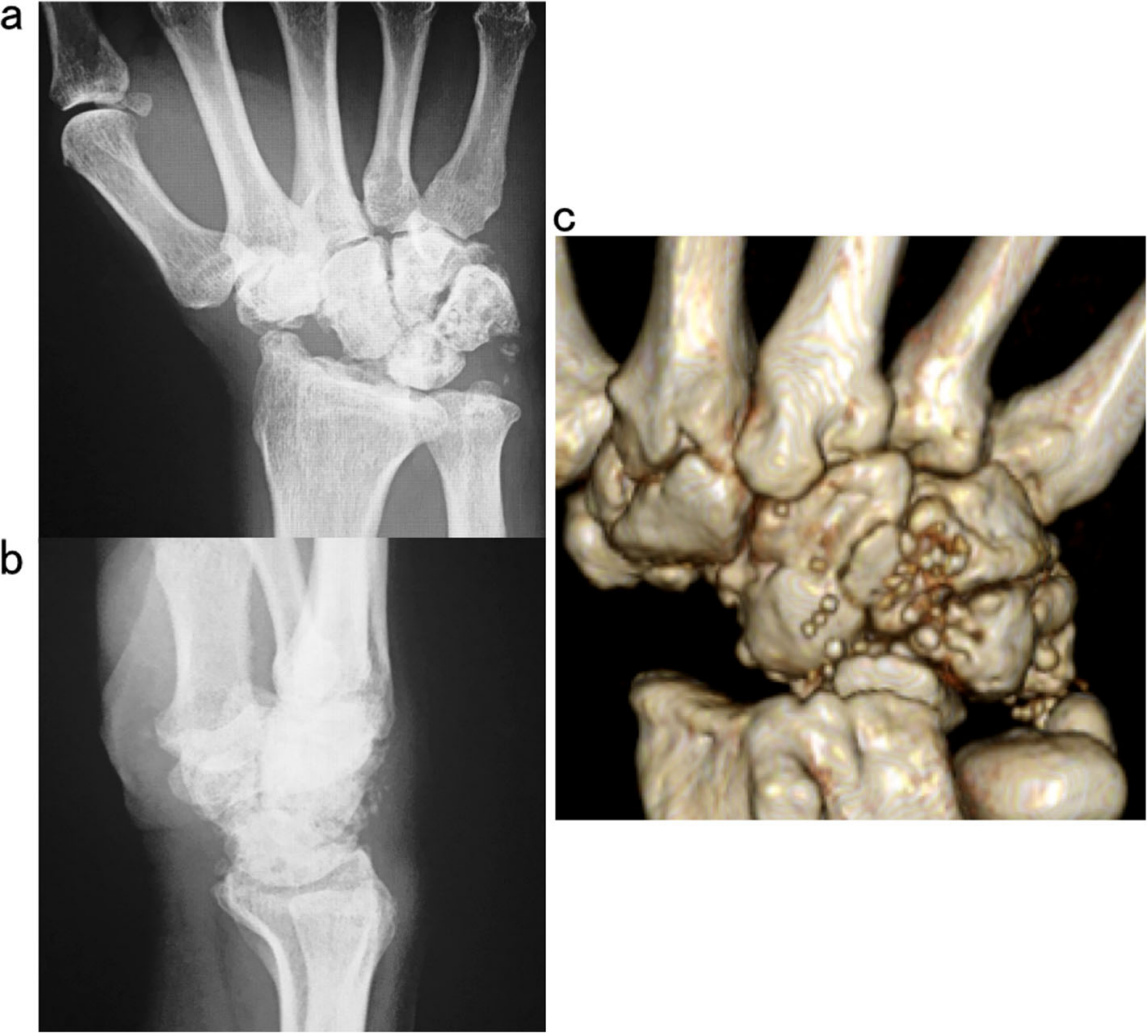
X-rays (**a**, **b**) and three-dimensional computed tomography scan. Fifty-six-year-old male patient 5 years after the operation. Note that the four corners have united and the BAP has almost disappeared. BAP, bioabsorbable plate

**Fig. 9:**
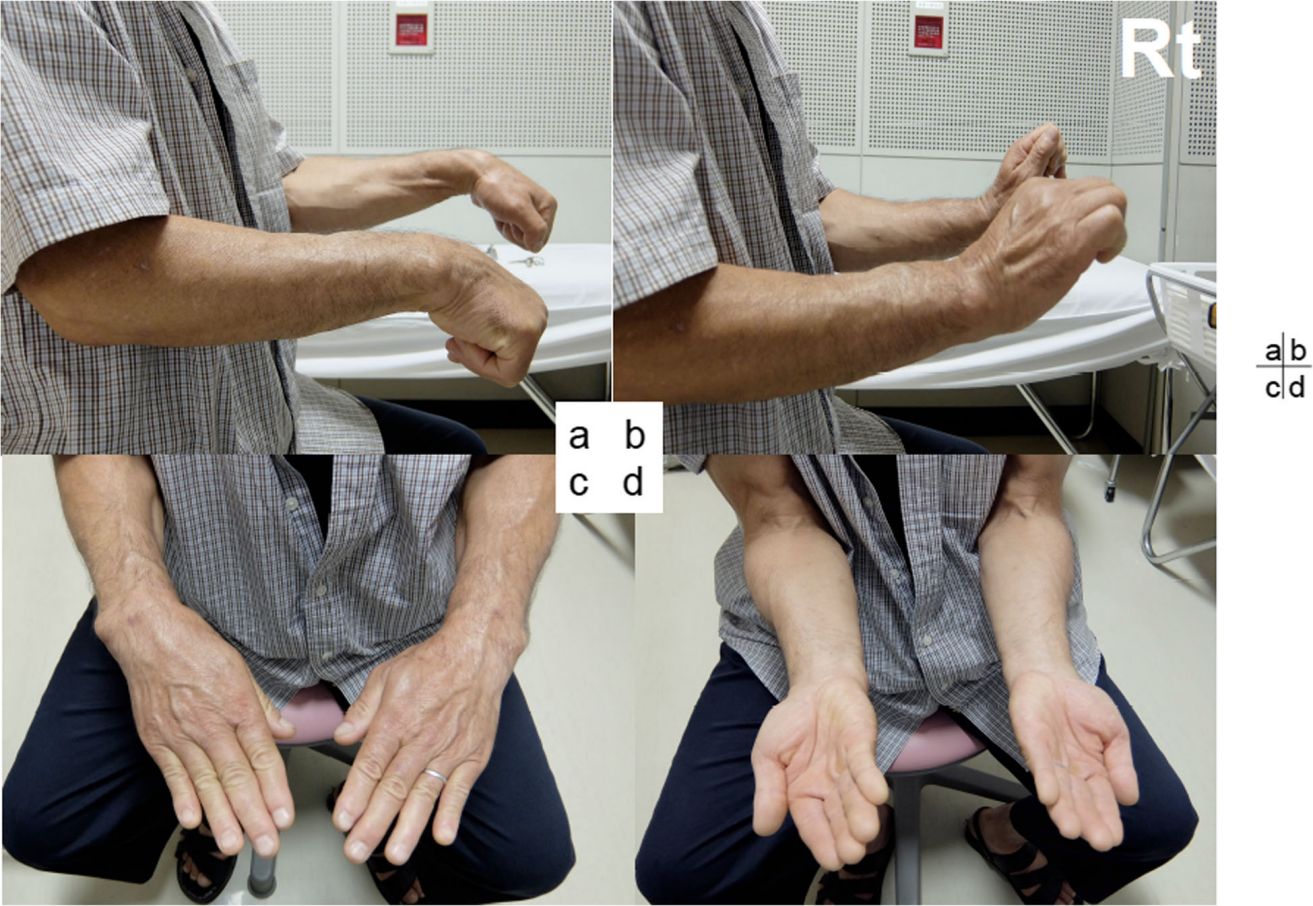
Clinical evaluation at 5 years. This shows 50° volar-flexion (**a**), 40° dorsi-flexion (**b**), 85° pronation (**c**), and 90° supination (**d**)

Case 1: A 56-year-old male farmer presented with radioscaphoid and capitolunate osteoarthritis of the right wrist following a fall 5 years prior. The GS was 74% and the *Quick*DASH score was 29.5. Due to wrist pain and arthritis, the patient was diagnosed as having SNAC stage III (Fig. 5a, b, c, d), as evaluated by an X-ray, computed tomography (CT) scan, and bone scintigram. The four corners were fixed using a circle-shaped BAP. After fixation, the joint capsule was sutured onto the BAP. The patient was assessed radiographically directly post operation (Fig. 6 a, b), and 1 year postoperatively via a three-dimensional CT scan (Fig. 7a, b, c). Five years after the operation, the four corners had united, and the plate had almost disappeared (Fig. 8a, b, c). At that stage, the active ROM was VF, 50°; DF, 40°; pronation, 85°; and supination, 90° (Fig. 9a, b, c, d). Radial bending was 25°, ulnar bending was 30°, GS was 25 kg (67%), the *Quick*DASH score was zero, and the level of pain was none.

**Fig. 10:**
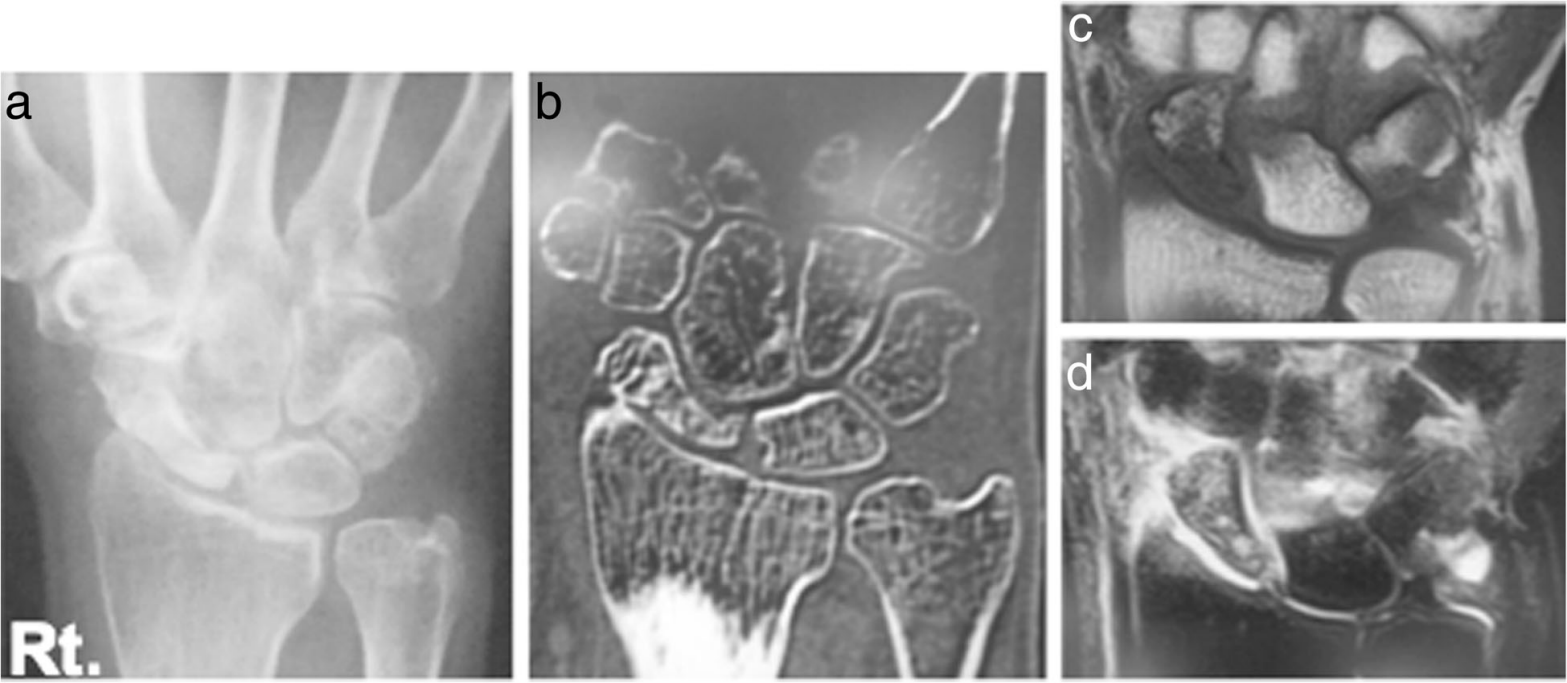
Radiograph (**a**), and magnetic resonance images (**b**, **c**, **d**). Sixty-one-year-old female patient with scaphoid nonunion advanced collapse stage III. No previous trauma was noted

**Fig. 11:**
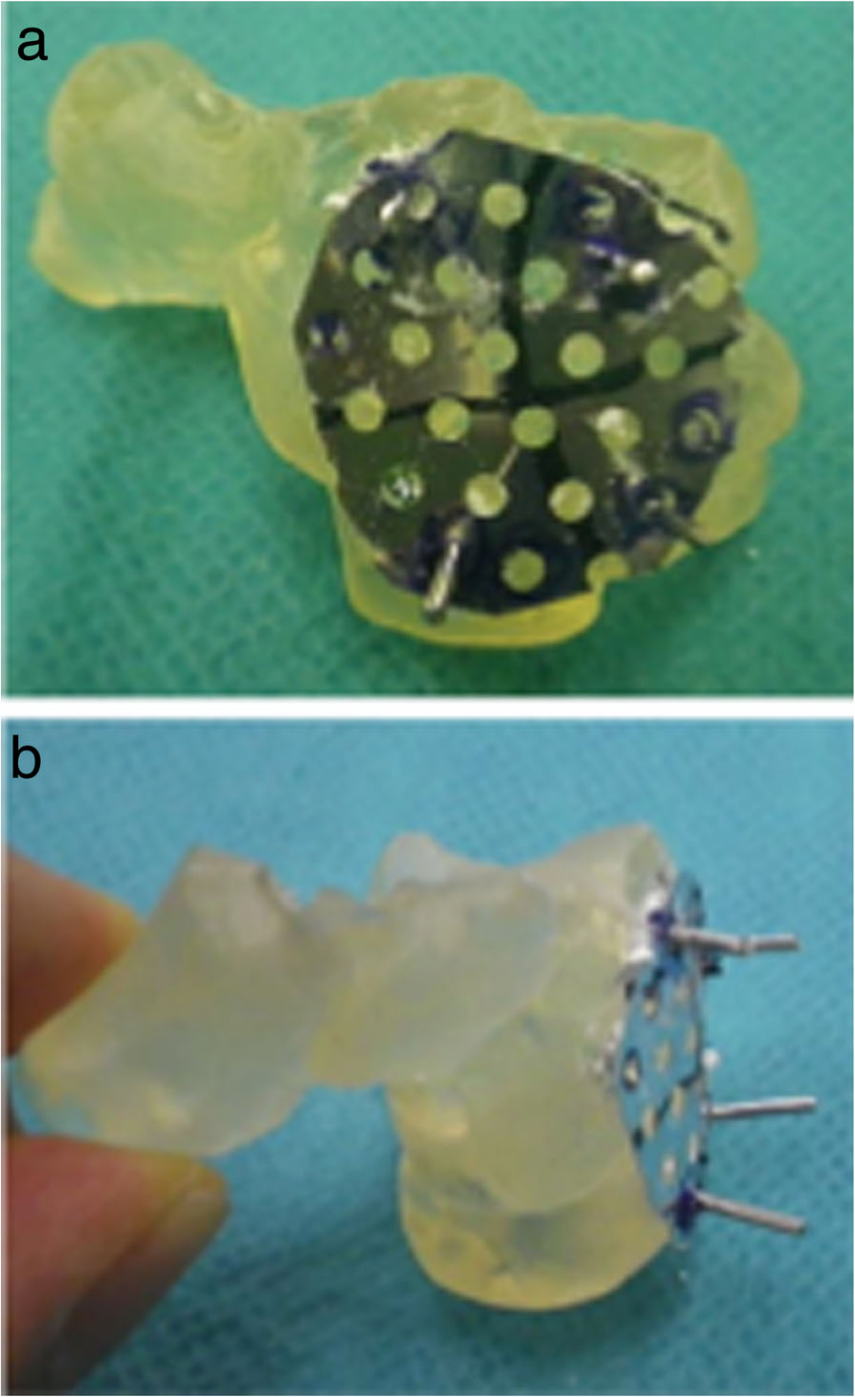
**a**, **b**: A three-dimensional printed model was created from the patient’s images. This was utilized to plan the appropriate form of the BAPs prior to surgery. BAP, bioabsorbable plate

**Fig. 12:**
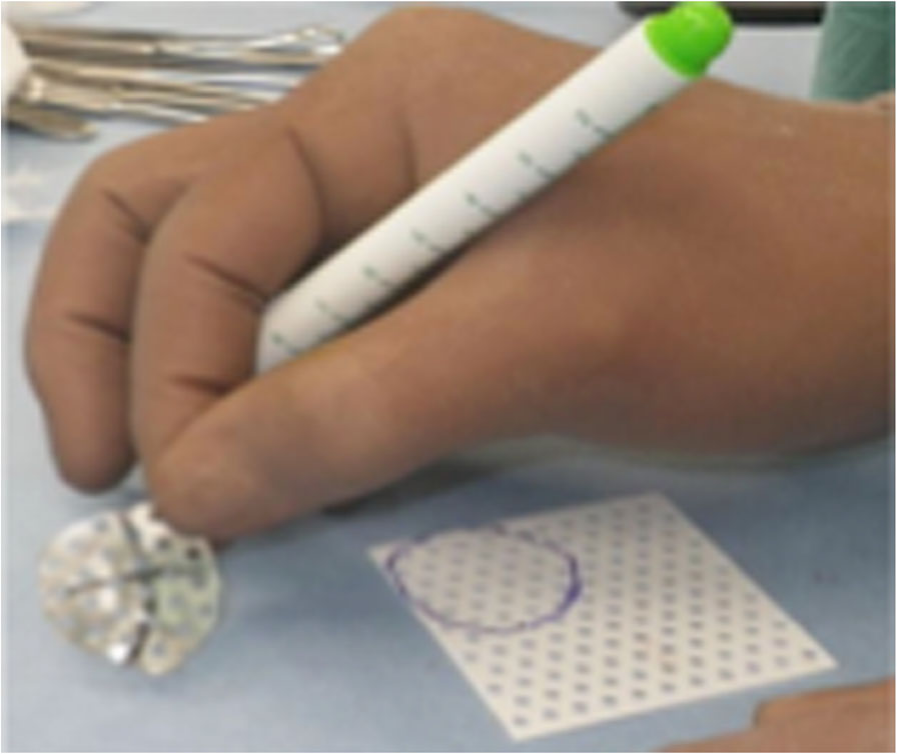
Preoperative planning of BAPs. BAP, bioabsorbable plate

**Fig. 13:**
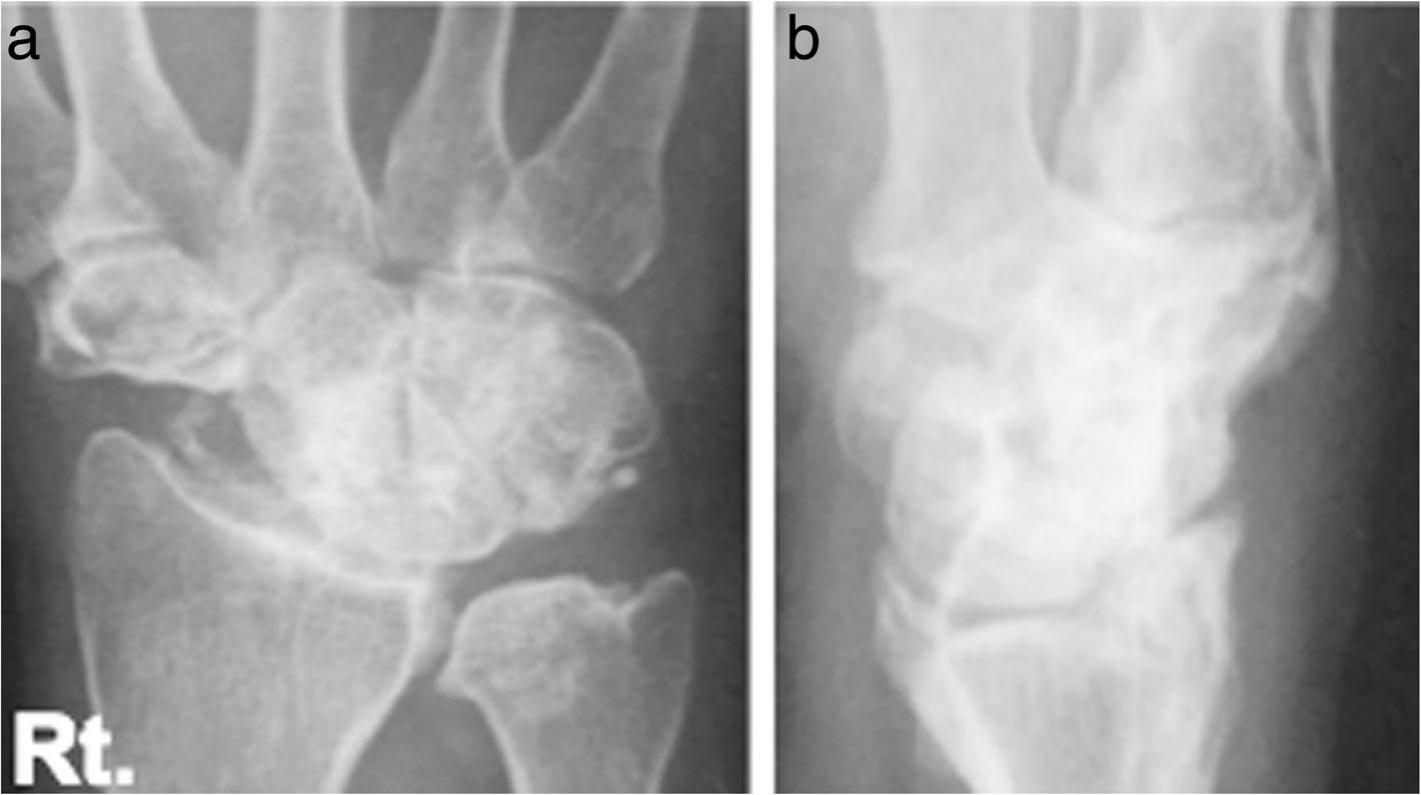
X-ray showing fully united (**a**, **b**) four corners

Case 2: A 61-year-old female with no obvious history of trauma presented with a SNAC stage III right wrist (Fig. 10a, b, c, d). Her GS was 78% and MRI results showed obvious scaphoid advanced collapse. In this case, as part of preoperative planning, a stereo model of the wrist was created using a three-dimensional printer. This was done to understand the appropriate shape of the BAP required for the surgery (Fig. 11a, b). In practice, the BAP is created during the surgery based on the results of the preoperative planning (Fig. 12). At 1.5 years post operation, the four corners were fully united (Fig. 13a, b); her GS was 21.9 kg (87%), *Quick*DASH score was 6.82, and the level of pain was none.

## Discussion

**Fig. 14:**
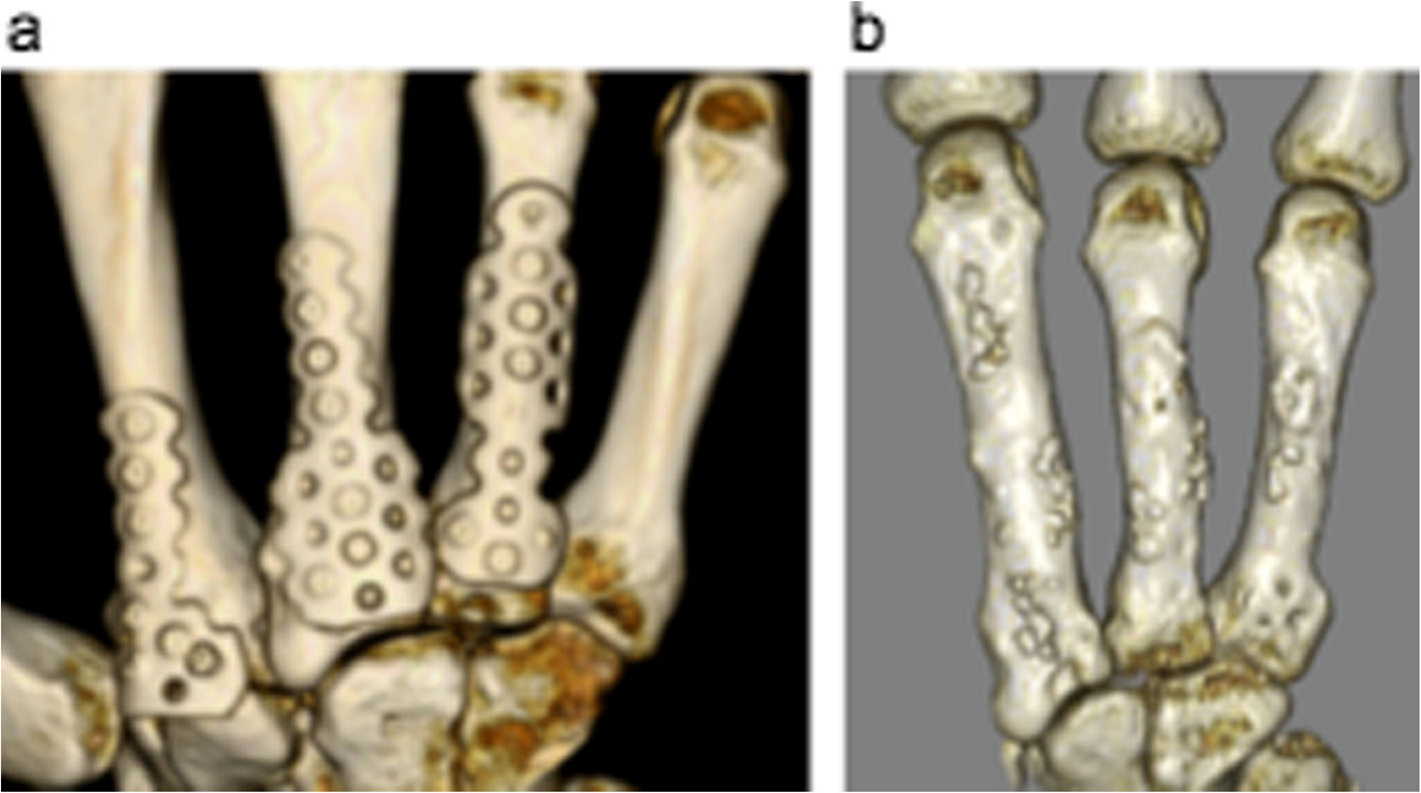
BAP shown on computed tomography scan (**a**) and is fully bioabsorbable (**b**). BAP, bioabsorbable plate

Absorbable implants are designed to provide several clinical advantages over metallic implants [11]. Firstly, mesh sheets made from u-HA/PLLA are freely moldable and can be placed in the best possible position to cover the projected area. Secondly, there are numerous screw holes in the mesh sheets; therefore, surgeons have more flexibility regarding screw placement. Thirdly, the material is visible on X-rays and CTs (Fig. 14a). Fourthly, the u-HA/PLLA material also serves as a good alternative for patients who are allergic to metal. Lastly, above all, a major advantage is that there is no need to remove the u-HA/PLLA BAP, as it is fully bioabsorbable (Fig. 14b).

In this study, the rate of bone union was 80% and patient satisfaction was 90%. We believe that these results are radiographically and clinically comparable to those of FCA using Xpode by Rudnick et al. [9].

This operative technique is uncomplicated and has provided us with tips from our experience in this study. We shaved the bone surface at the plate fixation area (1–2 mm depth) to inlay the crafted BAP. This promotes integration into the four corners and makes the construct extremely stable (Figs. 1, 2).

**Fig. 15:**
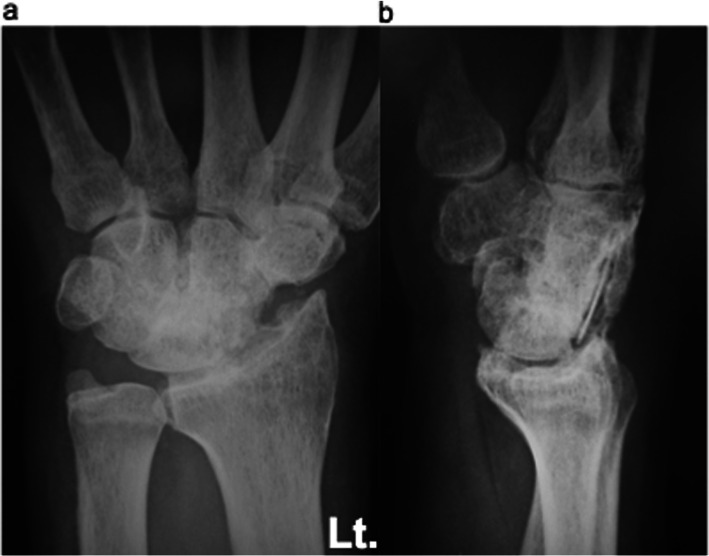
X-ray (**a**: AP view, **b**: Lateral view). This shows bone formation occurred onto the BAP 1-year post operatively. Preparing the bone (**a**) for the inlay of the BAP (**b**). BAP, bioabsorbable plate

Due to the highly osteoconductive nature of HA, an increased amount of bone forms on the BAP. This was clearly demonstrated in another case (Fig. 15a, b), where bone formation occurred onto the BAP 1-year post operatively.

## Conclusions

We summarized the outcomes of FCA using BAPs. Satisfactory clinical results were shown, with no obvious complications. This novel plate also serves as a good alternative for patients who are allergic to metals. Furthermore, BAPs are advantageous as they do not require removal.

## Supplementary information

**Additional file 1.** The procedure of four-corner fusion method using a bioabsorbable plate for scapholunate advanced collapse and scaphoid nonunion advanced collapse wrists

## Data Availability

The datasets used and/or analyzed during the current study are available from the corresponding author on reasonable request.

## Abbreviations

BAP: Bioabsorbable plate
CT: Computed tomography
DCP: Dorsal circular plates
DF: Dorsi-flexion
FCA: Four-corner arthrodesis
GS: Grip strength
*Quick*DASH: Quick Disabilities of the Arm, Shoulder, and Hand
ROM: Range of motion
SLAC: Scapholunate advanced collapse
SNAC: Scaphoid nonunion advanced collapse
VF: Volar-flexion
